# Supplementary material for: Mechanisms governing the pioneering and redistribution capabilities of the non-classical pioneer PU.1
Source: Nat Commun. 2020 Jan 21;11:402. doi: 10.1038/s41467-019-13960-2 (PMC6972792; doi:10.1038/s41467-019-13960-2)
Supplement: Supplementary file 7 — Source data [file 41467_2019_13960_MOESM7_ESM.zip › Source_Data/FigureS5/FigureS5A_MotifsCommonPeaks/homerResults/motif2.similar.html]

motif2

## Information for motif2

A
G
T
C
C
G
T
A
A
G
T
C
A
C
G
T
A
C
G
T
A
G
T
C
A
G
C
T
  
Reverse Opposite:  

C
T
G
A
A
C
T
G
C
G
T
A
C
G
T
A
A
C
T
G
A
C
G
T
C
T
A
G
  

|  |  |
| --- | --- |
| p-value: | 1e-717 |
| log p-value: | -1.653e+03 |
| Information Content per bp: | 1.938 |
| Number of Target Sequences with motif | 3595.0 |
| Percentage of Target Sequences with motif | 40.46% |
| Number of Background Sequences with motif | 4740.7 |
| Percentage of Background Sequences with motif | 12.47% |
| Average Position of motif in Targets | 99.6 +/- 54.7bp |
| Average Position of motif in Background | 99.1 +/- 74.5bp |
| Strand Bias (log2 ratio + to - strand density) | 0.0 |
| Multiplicity (# of sites on avg that occur together) | 1.08 |
| Motif File: | file (matrix) reverse opposite |

### Similar de novo motifs found

|  |  |  |  |  |  |  |  |
| --- | --- | --- | --- | --- | --- | --- | --- |
| Rank | Match Score | Redundant Motif | P-value | log P-value | % of Targets | % of Background | Motif file |
| 1 | 0.789 | T A G C C G T A G A T C A C G T G C A T T G A C G A T C G T A C | 1e-151 | -349.848939 | 13.12% | 4.84% | motif file (matrix) |
| 2 | 0.754 | C T G A A C T G C G T A C G T A A C T G A G C T C G T A A C T G | 1e-66 | -153.537394 | 9.26% | 4.36% | motif file (matrix) |
| 3 | 0.910 | G A T C G C A T G A T C G A T C G A T C C G T A A G T C A C G T A C G T G T A C A G C T T A G C | 1e-45 | -103.671916 | 3.33% | 1.08% | motif file (matrix) |
| 4 | 0.784 | C G T A A T G C G A C T A G C T A G T C A C G T A G T C A G C T C G T A G C A T | 1e-34 | -79.941492 | 3.14% | 1.17% | motif file (matrix) |
